# Psoriasis-associated vascular disease: the role of HDL

**DOI:** 10.1186/s12929-017-0382-4

**Published:** 2017-09-14

**Authors:** Maria Joao Paiva-Lopes, José Delgado Alves

**Affiliations:** 1 Serviço de Dermatologia, Hospital dos Capuchos CHLC, Alameda de Santo António dos Capuchos, 1169-050 Lisboa, Portugal; 2 CEDOC, NOVA Medical School | Faculdade de Ciências Médicas, Universidade NOVA de Lisboa, Campo dos Mártires da Pátria, 130, 1169-056 Lisboa, Portugal; 3 Immunomediated Systemic Diseases Unit (UDIMS), Fernando Fonseca Hospital, Amadora, Portugal

**Keywords:** Psoriasis, Atherosclerosis, High-density lipoproteins (HDL), HDL function, Anti-HDL antibodies

## Abstract

Psoriasis is a chronic inflammatory systemic disease with a prevalence of 2–3%. Overwhelming evidence show an epidemiological association between psoriasis, cardiovascular disease and atherosclerosis. Cardiovascular disease is the most frequent cause of death in patients with severe psoriasis. Several cardiovascular disease classical risk factors are also increased in psoriasis but the psoriasis-associated risk persists after adjusting for other risk factors.

Investigation has focused on finding explanations for these epidemiological data. Several studies have demonstrated significant lipid metabolism and HDL composition and function alterations in psoriatic patients. Altered HDL function is clearly one of the mechanisms involved, as these particles are of the utmost importance in atherosclerosis defense. Recent data indicate that biologic therapy can reverse both structural and functional HDL alterations in psoriasis, reinforcing their therapeutic potential.

## Background

Psoriasis is an immunomediated disease classified by some authors as autoimmune [1, 2], although the nature of the antigens involved is still unknown. It is the most prevalent chronic inflammatory disease in humans, with an estimated global prevalence of 2–3% [3]. Erythematous plaques covered by silvery scales characterize it clinically. Pathology shows keratinocyte hyperplasia and an inflammatory infiltrate. Psoriatic arthritis has been recognized as a comorbidity for many years but only recently has the association with other comorbidities been identified and studied [4]. Psoriasis patients have a higher prevalence of cardiovascular disease (CVD) and associated risk factors, including dyslipidaemia, higher blood pressure and higher body mass index, as demonstrated in a meta-analysis comprising 59 studies with up to 18,666 cases and 50,724 controls [5]. Furthermore, psoriasis is an independent risk factor for CVD even after controlling for traditional risk factors.

Cardiovascular disease is associated with a 6-year shorter life expectancy in patients with severe psoriasis [6]. According to a meta-analysis of 14 studies, the risk for cardiovascular mortality was 1.37, the risk for myocardial infarction was 3.04 and the risk for stroke 1.59 times higher in patients with severe psoriasis than in the general population [7].

Atherosclerosis is the pathological process underlying cardiovascular disease (CVD) and mortality. All phases of atherosclerosis are related to inflammatory events, in a strikingly similar fashion to the inflammatory events observed in psoriasis [8]. Furthermore, these two diseases share common immunologic pathways involving dendritic cells, Th1, Th17 and Treg cells as well as several angiogenic factors and oxidative mechanisms [8, 9]. Not surprisingly, psoriasis has been demonstrated to be an independent risk factor for subclinical atherosclerosis [10, 11].

High-density lipoproteins (HDL) are of pivotal importance in atherosclerosis. These are extremely complex particles with a unique capacity for milieu adaptation, constantly changing both its chemical composition and its tri-dimensional structure [12]. HDL has anti-oxidative, anti-inflammatory, anti-apoptotic and anti-thrombotic functions [13] hence its importance goes much beyond reverse cholesterol transport. These functions depend more on HDL quality and function than on HDL–C levels [14].

This review discusses psoriasis-associated alterations in HDL structure and function that may play a role in atherogenesis and vascular disease associated with psoriasis. We searched ISI Web of Science, Medline and PubMed for psoriasis, atherosclerosis, cardiovascular disease, HDL, dysfunctional HDL and anti-HDL antibodies. We started by searching for each term separately and afterwards an advanced search was carried out by combining “psoriasis” with each search term in keywords, abstracts and/or titles. The references cited in the selected articles were also analysed. After a comprehensive review, duplications were excluded and the final selection was based on relevance and date.

## Lipid metabolism in psoriasis

Lipid metabolism in patients with psoriasis has been a subject of study for more than 50 years. Numerous studies show decreased levels of HDL and/or increased levels of low-density lipoproteins (LDL) and of very-low density lipoproteins (VLDL) and triglycerides (TG) [15–18]. However, some studies failed to demonstrate an association between lipid serum levels and psoriasis [19–21], including a large population-based cross-sectional study in the UK [22]. In this study, psoriatic patients had hyperlipidaemia (odds ratio 1.16) but the results were not significant when controlled for obesity and diabetes, which can also influence lipids.

The published studies are heterogeneous because they include patients with different disease durations and systemic treatments, factors that may per se influence the lipid metabolism. Based on the results of 200 patients investigated at the onset of skin disease showing higher cholesterol concentrations in VLDL and HDL, Mallbris et al. [23] suggested that lipid abnormalities in psoriasis could be genetically determined rather than a consequence of the psoriasis pathogenic events and treatments on the lipoprotein phenotype. The lipid disturbances associated with the immunologic abnormalities led to the suggestion that psoriasis should be regarded as an immunometabolic disease [18].

## HDL composition and function

Lipoproteins form a complex lipid transport system. The smallest lipoproteins in molecular size are HDL and they contain the highest proportion of apolipoproteins to lipids. Apolipoprotein A-I (apoA-I) and apolipoprotein A-II (apoA-II) are major HDL associated proteins. They are secreted into plasma by the liver and the intestine. The highly dynamic and flexible conformation of the apoA-I molecule provides a possible explanation for how apoA-I determines HDL subclass structure and heterogeneity [12, 24, 25].

HDL takes up cholesterol from cell membranes and other lipoproteins and transports it from the periphery to the liver for excretion during the process of reverse cholesterol transport (RCT). Active export of excess cholesterol to HDL is mediated by the ATP-binding cassette transporters ABCA1 and ABCG1/G4. RCT is a process of major biological importance and is believed to be an important mechanism to explain how HDL might prevent cardiovascular disease.

Furthermore, HDL has several anti-inflammatory and antioxidant functions that may also be as important as RCT in protecting against atherosclerosis (Table 1). Noticeably, HDL has antioxidant properties, attributable mostly to apoA and paraoxonase (PON-1), and inhibits oxidative modifications of LDL. HDL also inhibits monocyte transmigration as well as the expression of adhesion molecules in endothelial cells. These properties seem to be independent from RCT [13, 26].

**Table 1:** Main atheroprotective HDL functions

HDL functions	
Cholesterol reverse transport	Cholesterol taken up from peripheral tissues, esterified and transferred to HDL for transport to the liver
Cholesterol efflux	Cholesterol and phospholipids removal from macrophages Interaction with cellular receptors: ABCA1, ABCG1, SR-BI
Anti-inflammatory activity	Inhibition of VCAM-1 expression in endothelial cells Removal of oxidized pro-inflammatory lipids from cells and lipoproteins
Antioxidative	Inhibition of LDL oxidation Removal of oxidized lipids from cells and ox-LDL Scavenging of ROS
Cytoprotective	Inhibition of endothelial cell apoptosis Efflux of toxic oxysterols
Vasodilatory	Induction of NO production Inhibition of superoxide release
Anti-thrombotic	Inhibition of platelet aggregation Activation of eNOS
Anti-infectious	Interaction with LPS

*HDL* high-density lipoproteins, *ABCA1* ATP-binding cassette transporter A1, *ABCG1* ATP-binding cassette transporter G1, *SR-BI* scavenger receptor class B type I, *VCAM-1* vascular cell adhesion molecule-1, *LDL* low-density lipoprotein, *ox-LDL* oxidized LDL, *ROS* reactive oxygen species, *NO* nitric oxide, *eNOS* endothelial nitric oxide synthase

Apolipoprotein E (apoE) is another important component of the HDL particle [27]. It is a polymorphic multifunctional protein recognized for its remarkable capacity to suppress atherosclerosis. Animal models show a non-lipid antiatherogenic effect of apoE, possibly explained by its antioxidant, antiproliferative (smooth muscle cells and lymphocytes), anti-inflammatory, antiplatelet, and nitric oxide (NO)–generating properties [28]. Furthermore, apoE suppresses nuclear factor-κB mediated inflammation in monocytes and macrophages, thus supressing atherosclerosis [29]. ApoE is also involved in cellular signalling and it may control macrophage plasticity and endothelial cell activation. Inflammatory cytokines can upregulate or downregulate the production of apoE in various tissue types and the cross-talk between apoE and cytokines has an important role in several disorders [30].

ApoE polymorphisms modulate susceptibility to many diseases, namely neurodegenerative disorders and atherosclerosis, as well as psoriasis [31, 32]. In a meta-analysis involving seven studies with 966 patients and 1086 controls, results indicate that the allele ε4 may influence psoriasis severity in Europeans, the allele ε2 is associated with increased susceptibility for psoriasis and the allele ε3 may decrease psoriasis risk [32]. These results must be interpreted with caution since the allelic distribution of apoE varies worldwide and the number of studies in the literature is small and involves only Europeans and Asians. Alleles ε2 and ε4 are also associated with increased cardiovascular risk [33].

Whilst most studies have been focused on the HDL protein components, the HDL lipids represent 50–80% of the total HDL mass and have been shown to affect particle stability, cholesterol efflux from macrophages and cholesterol elimination [34]. It is now recognized that lipid composition, namely phospholipids also play an essential role on HDL functionality [25, 34–36].

## HDL and chronic inflammation

HDL has known immunomodulating properties related both to the innate and the adaptive immune system [37].

HDL modulates several immune cell functions, mainly by modifying the lipid rafts’ cholesterol content. Thereby it modulates dendritic cells (DC), monocytes, macrophages, T cells and B cells [37]. Importantly, apoA-1 stimulates production of IL-10 and PGE2, thus inhibiting DC differentiation and function [38] and decreasing T cell activation and IL-12 production [39]. HDL protects from oxidative damage by terminating chain reactions of lipid peroxidation. Several apolipoproteins (apoA-I, apoA-II, apoA-IV, apoA-V, apoE, apoJ, apoM) and enzymes (PON1, LCAT, platelet activating factor-acetyl hydrolase) contribute to this effect [24].

In chronic inflammatory diseases however, the antioxidative and anti-inflammatory properties of HDL become impaired [40]. Proteome alterations, which include decreased activity of HDL-associated enzymes and accumulation of complement (C3), ceruloplasmin and SAA, result in loss of the antioxidant capacity of HDL.

Impaired cellular efflux of lipids to HDL is another pro-inflammatory mechanism that is associated with activation of intracellular STAT3 signalling and enhanced vascular inflammation [41].

These phenomena are important in chronic inflammatory diseases like rheumatoid arthritis and systemic lupus erythematosus (SLE), which are also associated with increased risk of cardiovascular disease. Chronic inflammation induced alterations in lipid metabolism certainly contribute to the higher atherosclerotic risk in these patients.

In patients with psoriasis, not only lipoprotein levels can be altered, but also their composition and function may be significantly different from controls. This is an important observation because the composition and function of HDL are actually considered more important than the quantity of HDL itself.

Mehta et al. [42] found a more atherogenic lipoprotein profile and decreased HDL efflux capacity in 112 psoriasis patients when compared to controls. In this study, nuclear magnetic resonance showed an atherogenic profile in psoriasis similar to that observed in diabetes, with significant increase in LDL particle concentration and decrease in LDL size. Also HDL efflux capacity was lower in psoriasis compared to controls.

HDL isolated from 15 psoriatic patients [35] revealed significant differences in the HDL-associated proteins: reduction of apoA-1 and apo-M and higher levels of acute-phase proteins such as serum amyloid A (SAA), prothrombin, α-1-antitrypsin and α-1-acid glycoprotein 1. The lipid composition of HDL was also altered in these patients; a decrease in the phospholipid and cholesterol content of HDL was observed. Additionally, this study showed a diminished cholesterol efflux capability that correlated with loss of apoA-1, phosphatidylcholine and sphingomyelin in HDL from psoriatic patients. However, Holzer reported no modification of HDL anti-oxidative activity, assessed by the capacity to inhibit dihydrorhodamine oxidation.

A recent study demonstrated that psoriasis associated HDL particle characteristics and function were also altered in 44 paediatric patients with psoriasis. These patients showed reduced cholesterol efflux capacity compared to age-matched controls, before and after adjusting for confounding variables, suggesting that reverse cholesterol transport defects start early in life even with low levels of chronic inflammation [43].

A cross-sectional study designed to characterize the biologic activities of plasma lipoproteins, involving 25 patients with psoriasis and 25 controls, showed lower plasma levels of HDL and reduced PON-1 activity. PON-1 activity was found to negatively correlate to disease severity, measured by PASI. Other HDL properties were also altered, namely a) protection against LDL oxidation, b) inhibition of TNF-α induced monocyte adherence to endothelial cells, c) prevention of oxidized low-density lipoprotein (ox-LDL)-induced monocyte migration and d) protection of endothelial cells from TNF-α induced apoptosis [44].

Reduced PON-1 activity was also demonstrated by Usta [45] in a cohort of 25 patients and Ferreti in a study of 23 patients [46]. However, this results were unconfirmed by other studies which found an increased [20] or not altered [35] PON activity. Interestingly, PON1 55 M allele is a risk factor for psoriasis: a case-control study of 100 patients with psoriasis demonstrated an association of this allele with an impairment of the antioxidant system and an abnormal lipid metabolism [47].

In chronic inflammatory conditions, protein structural modification and appearance of neo-epitopes may lead to autoantibody production and further HDL dysfunction. Elevated titres of autoantibodies against HDL (aHDL) and apoA-I have been demonstrated in diseases associated with an increased cardiovascular risk [48]. These antibodies have been recognized as a new biomarker for vascular disease in the context of autoimmune diseases [49]. We have recently demonstrated the presence of aHDL and aApo A-I antibodies in patients with psoriasis. These antibodies were associated with increased disease severity and may contribute to the pathogenesis of atherosclerosis in this condition [50].

It should be emphasized that published studies are heterogeneous regarding both clinical and laboratorial data. Patient selection includes different types of psoriasis (e.g. with or without arthritis) in various clinical stages with varying degrees of inflammation or remission and under different systemic therapies. Laboratory methodology is also variable; measuring HDL-cholesterol is not equivalent to measuring HDL particle number. NMR spectroscopy and ion mobility can be used to quantify HDL particles, however, these methods give different estimates of HDL particle concentration and size. Another problem is that all methods used to assess HDL subclasses measure only static concentrations with no assessment of the dynamic processes regulating HDL subclasses [24]. Despite these difficulties, the studies clearly show that psoriasis alters both the proteomic and lipid composition of HDL and these changes result in decreased cholesterol efflux capacity and reduced anti-inflammatory and antioxidant properties of HDL (Table 2). Furthermore, these findings have been corroborated by the observation that anti-psoriatic therapy, both conventional (specially systemic) and biologic, recovers HDL composition and function [51–54]. Noticeably, treatment with anti-TNF-α was associated with amelioration of myocardial dysfunction [55]. These studies suggest that adequate treatment, especially with biologic agents, can improve psoriasis patients’ cardiovascular risk.

**Table 2:** Main reported psoriasis-associated HDL alterations

Ref: number of subjects	HDL alterations
Mehta (42): 112 patients	Reduced cholesterol efflux capacity
Holzer (35): 15 patients	Reduced cholesterol efflux capacity Reduced apoA-I, apoA-II, apoM Reduced phospholipid (phosphatidylcholine and sphingomyeline) and cholesterol content Increased SAA, prothrombin, α-1-anti-trypsine, α-1-acid-glycoprotein 1 Not altered PON-1 activity
Holzer (51): 15 patients	Reduced cholesterol efflux capacity Reduced apoA-I and apoM Reduced phospholipid (phosphatidylcholine and sphingomyeline) and cholesterol content Increased SAA, prothrombin, α-1anti-trypsine, α-1-acid glycoprotein 1 Reduced PON-1 activity
He (44): 25 patients	Reduced PON-1 activity Reduced anti-inflammatory activity
Usta (45): 25 patients	Reduced PON-1 activity
Ferretti (46): 23 patients	Reduced PON-1 activity Increased lipid peroxidation
Toker (20): 30 patients	Increased PON-1 activity
Tom (43): 44 paediatric patients	Reduced cholesterol efflux capacity

*HDL* high-density lipoprotein, *apoA-I* apolipoprotein A-I, *apo-A-II* apolipoprotein A-II, *apoM* apolipoprotein M, *SAA* serum amyloid A, *PON-1* paraoxonase 1

## Conclusion

Inflammatory processes occurring chronically in psoriasis have an impact on HDL in many different ways resulting in proteomic and lipid composition alterations and consequently altered HDL function. These alterations help to explain the epidemiological evidence associating psoriasis to atherosclerosis and CVD, though further investigation on this subject is necessary. Importantly, anti-psoriatic therapy reverses these alterations. A better understanding of these mechanisms of disease will contribute to the development of novel biomarkers and of new therapeutic strategies.
